# Single-nucleotide resolution analysis of the transcriptome structure of *Clostridium beijerinckii *NCIMB 8052 using RNA-Seq

**DOI:** 10.1186/1471-2164-12-479

**Published:** 2011-09-30

**Authors:** Yi Wang, Xiangzhen Li, Yuejian Mao, Hans P Blaschek

**Affiliations:** 1 Department of Agricultural and Biological Engineering, University of Illinois at Urbana-Champaign, Urbana, IL 61801, USA; 2 Institute for Genomic Biology, University of Illinois at Urbana-Champaign, Urbana, IL 61801, USA; 3 Department of Animal Sciences, University of Illinois at Urbana-Champaign, Urbana, IL 61801, USA; 4 Department of Food Science and Human Nutrition, University of Illinois at Urbana-Champaign, Urbana, IL 61801, USA; 5 Center for Advanced Bioenergy Research (CABER), University of Illinois at Urbana-Champaign, Urbana, IL 61801, USA

## Abstract

**Background:**

*Clostridium beijerinckii *is an important solvent producing microorganism. The genome of *C. beijerinckii *NCIMB 8052 has recently been sequenced. Although transcriptome structure is important in order to reveal the functional and regulatory architecture of the genome, the physical structure of transcriptome for this strain, such as the operon linkages and transcript boundaries are not well understood.

**Results:**

In this study, we conducted a single-nucleotide resolution analysis of the *C. beijerinckii *NCIMB 8052 transcriptome using high-throughput RNA-Seq technology. We identified the transcription start sites and operon structure throughout the genome. We confirmed the structure of important gene operons involved in metabolic pathways for acid and solvent production in *C. beijerinckii *8052, including *pta*-*ack*, *ptb*-*buk*, *hbd*-*etfA*-*etfB*-*crt *(*bcs*) and *ald*-*ctfA*-*ctfB*-*adc *(*sol*) operons; we also defined important operons related to chemotaxis/motility, transcriptional regulation, stress response and fatty acids biosynthesis along with others. We discovered 20 previously non-annotated regions with significant transcriptional activities and 15 genes whose translation start codons were likely mis-annotated. As a consequence, the accuracy of existing genome annotation was significantly enhanced. Furthermore, we identified 78 putative silent genes and 177 putative housekeeping genes based on normalized transcription measurement with the sequence data. We also observed that more than 30% of pseudogenes had significant transcriptional activities during the fermentation process. Strong correlations exist between the expression values derived from RNA-Seq analysis and microarray data or qRT-PCR results.

**Conclusions:**

Transcriptome structural profiling in this research provided important supplemental information on the accuracy of genome annotation, and revealed additional gene functions and regulation in *C. beijerinckii*.

## Background

Solvents such as acetone, butanol and ethanol (ABE) produced through microbial fermentation represent important potential renewable fuels and chemicals [1]. *Clostridium acetobutylicum *and *C. beijerinckii *are among the prominent solvent-producing species. Although *C. beijerinckii *is phenotypically similar to *C. acetobutylicum*, the saccharolytic strains are phylogenetically distant from the amylolytic *C. acetobutylicum *ATCC 824 type strain [2]. *C. beijerinckii *exhibits a broader substrate range and optimum pH for growth and solvent production [3]; thus it may have greater potential for biosolvent production than *C. acetobutylicum*.

The genome of *C. beijerinckii *NCIMB 8052 was sequenced by the DOE Joint Genome Institute in 2007 (JGI project ID 3634512). The genome size is 6.0 Mb, which is 50% larger than that of *C. acetobutylicum *ATCC 824. The *C. beijerinckii *8052 solvent-producing genes are all located on the chromosome, as opposed to the location of these genes on a mega-plasmid in *C. acetobutylicum *824. Although transcriptome structural organization is important in order to reveal the functional and regulatory architecture of the genome, such annotation for the *C. beijerinckii *8052 genome is far from complete. Current genome annotation was made by computational analysis based on gene prediction algorithms. Although this allows for the determination of the complete set of gene loci and intergenic regions of the genome, it does not provide sufficient information concerning the transcriptional organization on a genome-wide level. For example, transcriptome structures such as operon linkages and transcript boundaries, etc. are not well understood and lack confirmation with experimental approaches. Next-generation high-throughput sequencing technology enabled us to obtain millions of cDNA reads simultaneously. In RNA-Seq analysis, these reads can be assembled against the genome sequence, and expression values calculated based on the reads mapped to genes. Deep sequencing of cDNA pool allowed us to study the bacterial transcriptome structure and gene expression at an unprecedented resolution and depth [4-8]. In this study, we used RNA-Seq technology to investigate *C. beijerinckii *8052 transcriptome structure at a single-nucleotide resolution. We identified the transcription start sites and operon structure throughout the genome. We confirmed the structure of important gene operons involved in metabolic pathways for acid and solvent production in *C. beijerinckii *8052. We defined important operons related to chemotaxis/motility, transcriptional regulation, stress response and fatty acids biosynthesis. We discovered 20 previously non-annotated regions with significant transcriptional activities and 15 genes whose translation start codons were likely mis-annotated. The results from this study significantly enhanced the accuracy of current genome annotation, and provided an essential reference point for other researchers working in related fields.

## Results and discussion

### Growth kinetics and ABE fermentation

**Figure 1:**
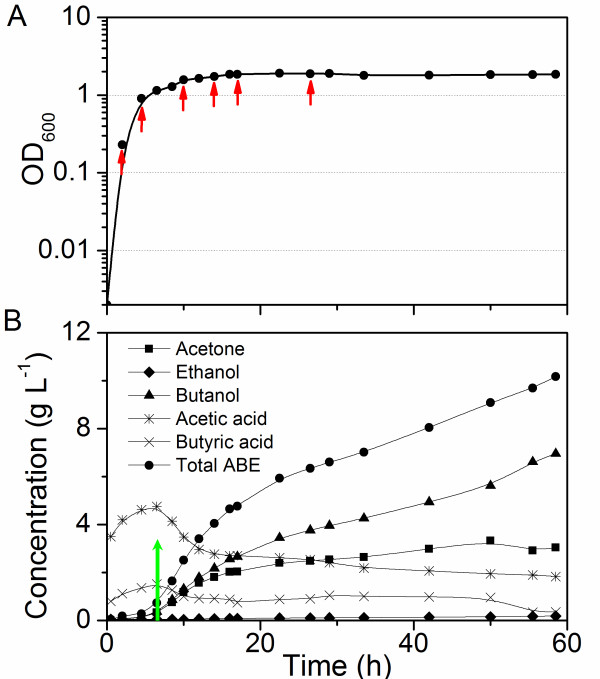
**Fermentation kinetics of *C. beijerinckii *8052 batch culture**. (A) Cell growth curve with sampling points for RNA-Seq indicated by red arrows. (B) Solvents and acids production over time with the onset of solvent production indicated by a green arrow.

*C. beijerinckii *8052 grew rapidly with a very short lag phase in the batch fermentation with P2 medium supplemented with yeast extract and glucose (Figure 1A). The fermentation experienced a shift from acidogenesis to solventogenesis at approximately 4.5-8 h. Formation of solvents was detected at between 4.5-6.5 h after the start of fermentation, which corresponded to the late exponential growth phase. Butanol continued to increase throughout the stationary phase (Figure 1B). Samples for RNA isolation were collected at time points during acidogenesis (2 and 4.5 h) and solventogenesis (after 6.5 h) (Figure 1A). The combined information from these samples collected at different growth phases is representative for transcriptome structural analysis.

### Transcriptome definition and structure

The 75-nt cDNA reads were mapped to the *C. beijerinckii *8052 genome. Only those reads that mapped unambiguously to the genome were used for further analyses (Table 1). Collectively, around 75.5% of the *C. beijerinckii *8052 genome was transcribed in at least one sample, even though the fraction in each single sample was much less.

**Table 1:** Summary of RNA-Seq sequencing and data analysis results

Sample	1	2	3	4	5	6*	Total
Time collected (h)	2	4.5	10	14	17	26.5	
Total No. of reads	8988633	9457480	8011531	8448929	10363535	38574501	83844609
No. of reads mapped	8473125	9037616	7514804	7730815	9842491	37676913	80275764
No. of reads unambiguously mapped	6776544	7274568	6096405	6189652	8096169	35027722	69461060
No. of bases unambiguously mapped	508240800	545592600	457230375	464223900	607212675	2627079150	5209579500
Percentage of genome represented	38.71	36.59	43.84	40.60	41.34	50.67	75.46
No. of genes with detectable expression**	4219	4082	4496	4453	4487	4750	5024
Range in expression levels (RPKM)	3.2 × 10^-1^ ~ 2.5 × 10^4^	5.8 × 10^-2^ ~ 6.0 × 10^4^	4.5 × 10^-2^ ~ 2.5 × 10^4^	7.0 × 10^-2^ ~ 9.0 × 10^4^	1.0 × 10^-1^ ~ 8.6 × 10^4^	3.6 × 10^-2^ ~ 9.8 × 10^4^	3.6 × 10^-2^ ~ 9.8 × 10^4^

*Sample 6 was sequenced with 1 sample/lane, while samples 1-5 with 2 samples/lane. See Methods section for more details; **Pseudogenes included.

The sequence coverage per base was plotted and visualized using the genome browser Artemis and DNAPlotter [5,9,10] (Additional file 1 Figure S1). The sequence coverage of the genes (based on number of mapped reads) for different samples was observed to be different in the overall sequence coverage profiles, which might be a reflection of the physiological states as the cell transited from one growth phase to another. For example, sample 2 represented a time period that was at the beginning of the transition from acidogenesis to solventogenesis. In this sample, genes encoding butyrate kinase (*buk*, 233080-234147 nt; Gene ID is listed in Additional file 2 Table S1, similarly hereinafter), acetyl-CoA acetyltransferase (*thl*,499121-500302 nt), glyceraldehyde-3-phosphate dehydrogenase (*gap*, 710763-711764 nt), fructose-bisphosphate aldolase (*fba*, 2199060-2199926 nt), aldehyde dehydrogenase (*ald*, 4399026-4400432 nt), acetoacetate decarboxylase (*adc*, 4401916-4402656 nt) were all actively expressed (high peaks in Additional file 1 Figure S1). These genes are involved in acid and butyryl-CoA formation, solventogenesis and glycolysis. The time frame of sample 4 was consistent with the transition to non-active growth and clostridial spores formation. In this sample, genes encoding stage V sporulation protein T (*spoVT*, 115288-115836 nt), spore coat protein cotJC (*cotJC*, 2411617-2412183 nt), spore coat peptide assembly protein cotJB (*cotJB*, 2412190-2412453 nt), small acid-soluble spore protein, sspA (*sspA*, 3596975-3597184 nt), small acid-soluble spore protein, sspC2 (*sspC2*, 3814296-3814505 nt) were actively expressed among others (Additional file 1 Figure S1). These genes are involved in the regulation of spore coat assembly and other sporulation-related processes [11,12]. These results were in good agreement with our previous observation using a 500-gene set DNA microarray [13]. A more detailed genome-wide transcriptional analysis of *C. beijerinckii *8052 during the shift from acidogenesis to solventogenesis is currently underway in our lab.

The distinct transcript boundaries and multigene operon structure in a genome are essential for elucidating a genome's regulatory mechanisms. Through transcriptome profiling analysis, the transcript boundaries and operon structures were identified across the entire *C. beijerinckii *8052 genome (Additional file 3 Table S2). Of all the > 5000 genes, 2151 genes (42.2%) were determined as being parts of multi-gene operon structures. The largest number of genes in a single operon was 32 (ribosomal proteins operon, Cbei_0150-0181, 192128-207934 nt). A refined genome annotation file (in GenBank format) was generated (Additional file 4) based on the findings from this work and the current *C. beijerinckii *8052 genome annotation in NCBI. The GenBank file can also be downloaded from https://netfiles.uiuc.edu/blaschek/www/Wang-BMC2011.

In addition, 5'-untranslated regions (5'-UTRs) were determined at the same time. Similar to other bacteria [4,14], most of the identified 5'-UTRs were very short. Among all the 1605 5'-UTRs that were estimated with high confidence (that is, valid 5'-UTRs identified in the 6 samples with a ≤ 50 nt window), 1262 were ≤ 50 nt, and only 65 were ≥ 100 nt (Additional file 5 Table S3). RibEx [15] was used to test for the putative regulatory elements among the 5'-UTRs over 100 bp. Three Predicted Riboswitch-like Elements (do not belong to any Rfam [16]; yet present very significant associations with either a COG or a KEGG pathway) were found in the 5'-UTRs of Cbei_0465, Cbei_1518 and Cbei_3542, respectively. In addition, a Known Riboswitch-like Element (flavin mononucleotide (FMN) riboswitch) was found in the 5'-UTR of Cbei_1224. More details about the identified putative riboswitches were described in Additional file 6 Table S4.

**Figure 2:**
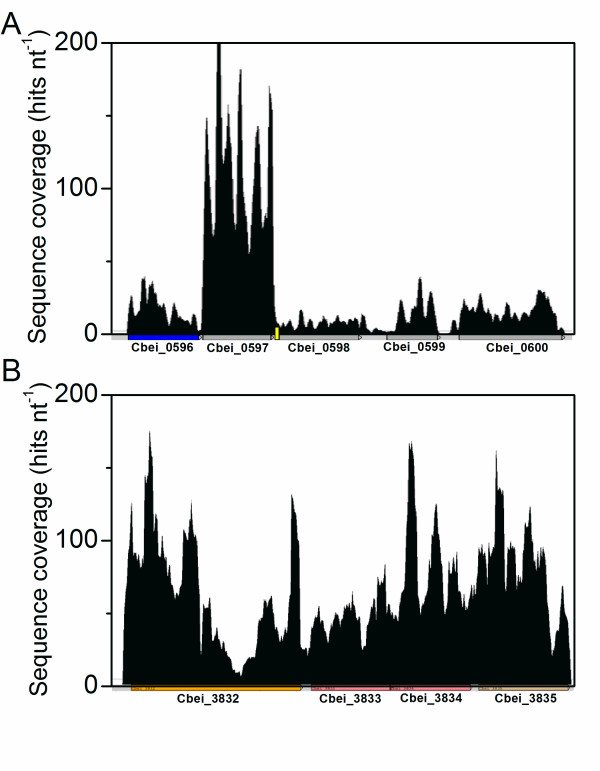
**Single-nucleotide resolution-sequencing data revealed important operon structures in *C. beijerinckii *8052**. (A) The glycolytic genes (*gap-pgk-tpi*) operon structure (from sample 1). The upstream gene Cbei_0596 encoding a putative transcriptional regulator protein was also linked with this operon. The intrinsic terminator predicted with TransTerm [18] is represented with a small yellow bar. (B) The solventogenic genes are organized in the *sol *operon in the order of *ald*-*ctfA*-*ctfB*-*adc*. Gene positions and directions are shown beneath by arrows. The color of arrows indicates the COG functional class of genes as defined in Additional file 1 Figure S1.

Previously, an operon containing genes encoding glyceraldehyde-3-phosphate dehydrogenase (*gap*), phosphoglycerate kinase (*pgk*) and triosephosphate isomerase (*tpi*) was revealed by transcriptional analyses for *C. acetobutylicum*, while the gene encoding phosphoglycerate mutase (*pgm*) was identified as not a member of this operon [17]. In this study, employing the single-nucleotide resolution sequence data, a similar *gap-pgk-tpi *operon was observed, upstream and apart from *pgm *for *C. beijerinckii *8052. In addition, it is very interesting that the upstream gene Cbei_0596 was also found to be linked with this operon (Figure 2A), which was validated by end-point RT-PCR as discussed below (Additional file 7 Figure S2 and Additional file 8 Table S5). The upstream gene (CA_C0708) of *gap *in *C. acetobutylicum *824 genome is conjectured to be the counterpart of Cbei_0596 in *C. beijerinckii *8052, since they both encode putative transcriptional regulator proteins and share 64% sequence identity. However, the intergenic distance between CA_C0708 and CA_C0709 in *C. acetobutylicum *824 genome is 146 nt, while that between Cbei_0596 and Cbei_0597 in *C. beijerinckii *8052 genome is only 71 nt. Although Cbei_0596-0599 are organized in the same operon, the sequence coverage depth downstream of *gap *was unexpectedly lower than that of the anterior (Figure 2A). An intrinsic terminator was predicted adjacent and downstream of *gap *(711818-711852 nt) using the bacterial genome transcription terminator prediction software TransTerm [18]. The terminator may have played a key role in attenuating the transcription of the downstream genes in this operon. Further experiments need to be carried out to confirm the activity and function of this element.

The butyryl-CoA formation related genes in *C. acetobutylicum *824 encoding 3-hydroxybutyryl-CoA dehydrogenase (*hbd*), crotonase (*crt*), and butyryl-CoA dehydrogenase (*bcd*) were identified as a *bcs *(butyryl-CoA synthesis) operon in the order of *crt*-*bcd*-*etfB *(encoding electron transfer flavoprotein subunit beta)-*etfA *(encoding electron transfer flavoprotein subunit alpha)-*hbd *[19,20]. A homologous operon structure was observed for *C. beijerinckii *8052 (Cbei_0321-0325) transcribed in the same order but the opposite direction (Additional file 3 Table S2). In addition, based on the sequencing data, similar *pfk *(phosphofructokinase)-*pyk *(pyruvate kinase), *pta *(phosphotransacetylase)-*ack *(acetate kinase), *ptb *(phosphate butyryltransferase)-*buk *gene operons were identified in *C. beijerinckii *8052 as those described in *C. acetobutylicum *824 [21-23] (Additional file 3 Table S2). Among them, *ptb-buk *operon was also verified by end-point RT-PCR experiment as discussed below (Additional file 7 Figure S2 and Additional file 8 Table S5).

Nearly all the genes encoding solventogenic enzymes have been cloned and characterized in *C. acetobutylicum *[24]. In *C. acetobutylicum *824, the genes *adhE *(acetaldehyde-CoA/alcohol dehydrogenase), *ctfA *(acetoacetyl-CoA: acetate/butyrate-CoA transferase subunit A) and *ctfB *(acetoacetyl-CoA: acetate/butyrate-CoA transferase subunit B) are located in the *sol *(solvent formation) operon on the mega-plasmid pSOL1 whose loss leads to the degeneration of the strain [25], while *adc *(acetoacetate decarboxylase) is organized in a monocistronic operon in the opposite direction [26,27]. In this study, a *sol *operon organized in the order of *ald*-*ctfA*-*ctfB*-*adc *was observed (Figure 2B and Additional file 3 Table S2). Previously, Chen and Blaschek (1999) speculated that the *ald*, *ctfA*, *ctfB *and *adc *genes were located in an operon following a Northern hybridization analysis of *C. beijerinckii *8052 total RNA [28]. With direct sequencing data, this study successfully confirmed the above hypothesis, and identified the transcriptional start sites (TSS) upstream of *ald *gene. This solventogenic gene arrangement in *C. beijerinckii *8052 is consistent with that observed in *C. beijerinckii *NRRL B593 and *C. saccharoperbutylacetonicum *N1-4 [29,30].

A flagellar/chemotaxis gene operon (CA_C2225-C2215) was previously defined in *C. acetobutylicum *[31]; a counterpart organized in exactly the same order (Cbei_4312-4302) in *C. beijerinckii *8052 was observed in this study (Additional file 3 Table S2). Similarly to *C. acetobutylicum *824, the transcriptional regulator *sigF *operon was also confirmed in *C. beijerinckii *8052 with the sequencing data that includes the forespore-specific sigma factor gene *sigF*, the anti-sigF factor gene *spoIIAB*, and the anti-anti-sigF factor gene *spoIIAA *(Additional file 3 Table S2). In *Bacillus subtilis *and *C. acetobutylicum*, the class I heat shock genes are organized in *dnaKJ *(organized in the order of *hrcA*-*grpE*-*dnaK*-*dnaJ *in *C. acetobutylicum *824) and *groESL *(*groES*-*groEL*) operons [32,33]. The similar organization of *dnaKJ *and *groESL *operons for *C. beijerinckii *8052 was also observed in this study (Additional file 3 Table S2). The fatty acid biosynthesis genes are organized in a single *fab *operon in *C. acetobutylicum *(CA_C3568-C3580) [34], while in this study, the *fab *genes (Cbei_1067-1077) in *C. beijerinckii *8052 were observed to be organized in four operons, which is similar to those of *B. subtilis *[35] (Additional file 3 Table S2).

**Figure 3:**
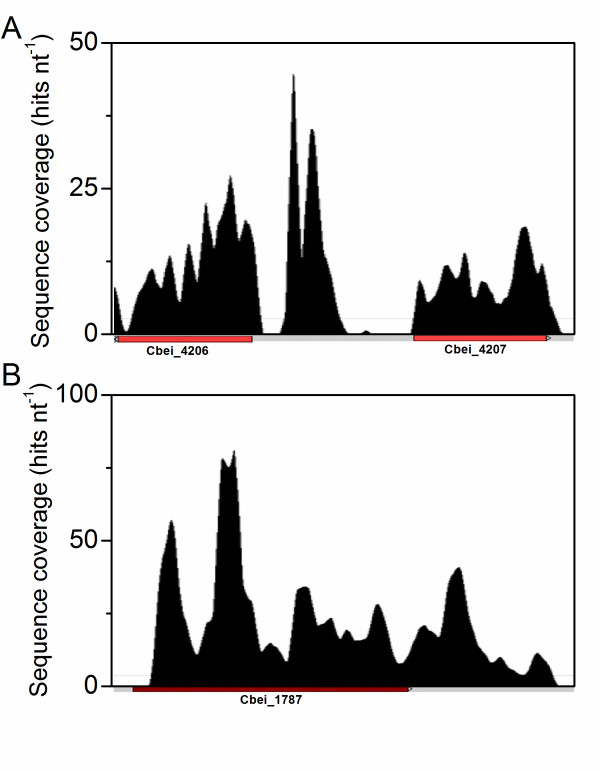
**Mapping the high resolution RNA-Seq data back to the genome allows the test of current *C. beijerinckii *8052 genome annotation**. (A) Sequence coverage in the Cbei_4206-4207 region, showing transcription activity across a region not included in the current genome annotation (from sample 2). (B) Sequence coverage near Cbei_1787, with the sequence coverage starts downstream the annotated translation start codon (from sample 5). Gene positions and directions are shown beneath by arrows. The color of arrows indicates the COG functional class of genes as defined in Additional file 1 Figure S1.

While the current *C. beijerinckii *8052 genome was annotated based on bioinformatical predictions, the RNA-Seq sequencing approach provides additional experimental evidence for genome annotation. By comparing the sequence coverage data to the genome annotation, 20 non-annotated regions were found to have significant transcriptional activities (Figure 3A, see also the supplemental texts in Additional file 9). These regions may represent potential new genes or regulatory RNAs. Twelve potential new genes were predicted in these regions using GeneMark [36]. Additional details about this test and the predicted genes were summarized in Additional file 10 Table S6.

In addition, 15 transcripts were identified with TSS that are downstream of the current annotated translation start sites (Figure 3B, see also the supplemental texts in Additional file 9). For these regions, re-annotation is needed since the current start codons may have been mis-annotated.

### Putative silent genes

Based on RNA-Seq sequence data, 78 protein-encoding genes demonstrated no transcripts over all six sampling time points, and these genes are likely silent (Additional file 11 Table S7). Thirty-one out of them were genes encoding hypothetical proteins. In addition, half of the genes encoding transposases (14 out of the total 29 in the genome) were silent. Although transposases may have played important roles during the evolution of *C. beijerinckii*, most of them are not as functional any more during the course of a batch fermentation. Besides, several genes encoding the subunits (such as soborse-specific subunits, lactose/cellobiose-specific subunits) of phosphotransferase system (PTS) related to the transport of sugars other than glucose were also among the list of silent genes. Since glucose was the only carbohydrate used in this study, these enzymes were not induced during the fermentation process.

### Putative housekeeping genes (HKGs)

Some genes have little variation in expression level through the entire fermentation process, and they are regarded as putative housekeeping genes (HKGs). For accurate gene expression quantification, normalization of gene expression against HKGs (endogenous control or reference gene) is generally required. In this study, 177 protein-coding genes were identified as putative HKGs with the lowest coefficient of variation (CV) in RPKM (see Methods section) values among all the sampling points (CV = standard deviation/mean; < 30% for listed genes in Additional file 12 Table S8) [37]. A COG functional group analysis by Fisher's exact test found that COG functional category D (Cell cycle control, mitosis and meiosis, 10.3%), L (Replication, recombination and repair, 9.2%) and E (Amino acid transport and metabolism, 6.6%) were overrepresented in this list [37,38]. This list of putative HKGs was generated based on the transcription data obtained from the six samples under the certain batch fermentation conditions employed in this study. This list can be considered as a starting point for identifying HKGs for *C. beijerinckii*. However, whether these genes are stably expressed under different experimental conditions requires further study.

A prediction of the promoters for primary sigma factors for all the putative HKGs was carried out using BPROM (http://linux1.softberry.com/berry.phtml). In total, 88 promoters along with their corresponding -10 box and -35 box sequences were predicted (Additional file 12 Table S8).

### Pseudogenes

Pseudogenes in bacteria genomes are non-functional copies of gene fragments usually created by random mutations and chance events [39,40]. Although pseudogenes are usually non-functional, it has been reported that quite a few of them can still go through the transcription process [41-43]. In this study, 26 out of the 82 pseudogenes in *C. beijerinckii *8052 genome were found to have significant transcriptional activities over the course of fermentation (Additional file 13 Table S9). However, although globally the 82 pseudogenes comprise about 0.6% of the predicted CDS of the genome, only < 0.1% of all the RNA-Seq reads mapped to these genes, indicating a significantly lower transcriptional activity when compared to the regular protein coding genes. In addition, six pseudogenes were found to be completely silent throughout the fermentation process (Additional file 13 Table S9). This is overrepresented among the silent genes when compared to the protein-coding genes based on a Fisher's exact test (*p *= 0.0015).

When pseudogenes share high sequence identity with other functional genes in the genome, it is usually on one hand very difficult to design probes to detect the transcription of pseudogenes with traditional methods, and on the other pseudogenes can lead to amplification bias during genetic studies of the functional genes with high sequence identity to pseudogenes [42]. Apparently such problems can be avoided with RNA-Seq method, which is one of the unprecedented advantages of RNA-Seq technique.

For summarization and easy reference to the readers, a table (Additional file 14 Table S10) summarizing the various supplementary information provided by RNA-Seq study to the current genome annotation was listed in the supplemental materials.

### End-point RT-PCR

End-point RT-PCR was used to validate the operon structure results obtained from RNA-Seq analysis [4]. One gene pair that is highly likely to be co-operonic with an intergenic distance of 7 bp was chosen as a positive control (Cbei_0341-0342). Ten other gene pairs with long intergenic distances (71-440 bp) where there were high transcription levels were chosen. All the operon structures determined by sequence data in the chosen gene pairs were confirmed by end-point RT-PCR results, indicating that RNA-Seq is a valid approach for operon structure identification (Additional file 7 Figure S2 and Additional file 8 Table S5). Among them, the *ptb-buk *operon and the linkage between Cbei_0596 and Cbei_0597 (*gap*) as discussed above were also confirmed.

### Correlations of RNA-Seq data with microarray and qRT-PCR data

**Figure 4:**
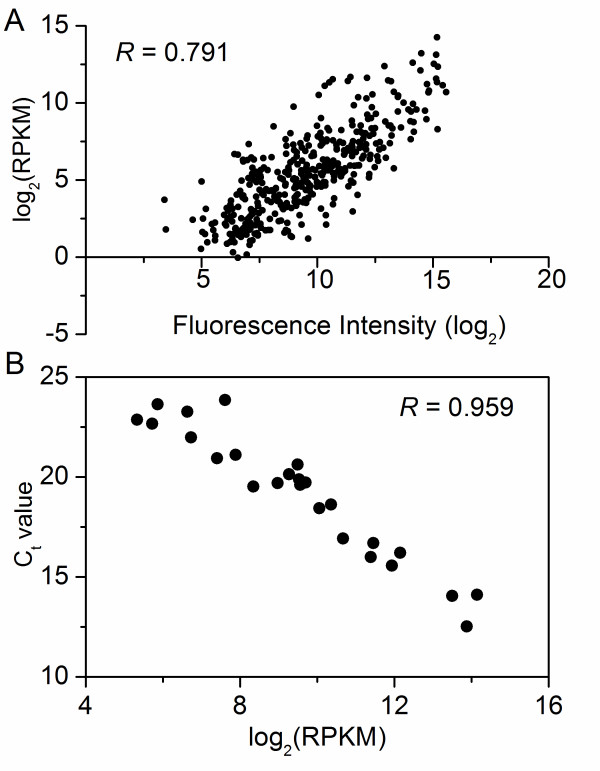
**Correlations of RNA-Seq data with microarray and qRT-PCR data**. (A) Comparison of normalized sequence coverage depth from RNA-Seq and absolute microarray fluorescence intensity. Shown is a plot of log_2_-transformation of RPKM for sample 2 (at 4.5 h) and raw microarray intensities for an equivalent sample (at 5 h) from Shi and Blaschek [13]. (B) Real time qRT-PCR verification of RNA-Seq results for selected genes (from sample 4, see also Additional file 15 Table S11).

**Figure 5:**
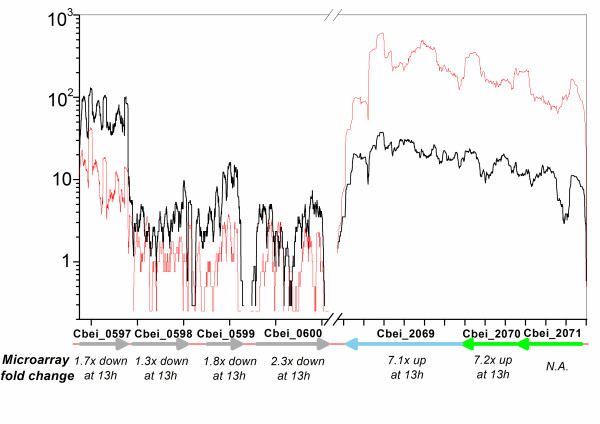
**Comparison of transcriptional profiles for sample 2 (4.5 h, black line) and sample 4 (14 h, red line), normalized to the gene length and genome-wide total number of unambiguously mapped reads for that sample**. Shown are an ~5.4 kb segment surrounding the *gap-pgk-tpi *glycolytic gene operon (Cbei_0597-0600) and an ~1.2 kb segment surrounding the *cotJC-cotJB *(Cbei_2069-2071) sporulation gene operon. Specific genes are indicated with arrows, whose colors indicate the COG functional class of genes as defined in Additional file 1 Figure S1. Below each gene is the expression level fold change that was measured by microarray data for equivalent samples (5 h vs. 13 h in Shi and Blaschek [13]; microarray data for Cbei_2071 are not available).

The RNA-Seq data obtained in this study were compared to the results obtained previously using a 500-gene set DNA microarray [13]. A small number of genes that did not allow for unambiguous mapping in RNA-Seq analysis were not included in the comparison. A good correlation was observed between the normalized coverage depth for sample 2 (collected at 4.5 h) and the raw microarray fluorescence intensities for an equivalent sample (collected at 5 h under the same growth condition) (Figure 4A). Specifically, the gene expression patterns of glycolytic genes Cbei_0597-0600 and sporulation genes Cbei_2069-2071 from samples 2 (4.5 h) and 4 (14 h) measured by sequencing data were compared. Similar differential expression patterns by microarray data from equivalent samples (5 h vs. 13 h) were observed (Figure 5). Although only a limited number of genes were compared, the good correlation between two methods further demonstrated the effectiveness of RNA-Seq approach. An additional advantage of RNA-Seq is that the sequence data can measure expression levels for every gene without bias caused by sequence-specific differences in hybridization efficiency in microarray-based methods [4,44], and sequence data allow one to measure gene expression more accurately with a much higher dynamic range as indicated in this study (Table 1) and other references [4,8,44,45].

The expression measurement with RNA-Seq data was further validated using real time quantitative reverse transcription PCR (qRT-PCR). Twenty-five genes were selected for the test (Additional file 13 Table S9). A high degree of correlation (*R *= 0.959) between the threshold value (Ct) and the log_2_-tranformation of RPKM was observed (Figure 4B).

## Conclusions

A single-nucleotide resolution analysis of the *C. beijerinckii *NCIMB 8052 transcriptome structure was conducted using high-throughput RNA-Seq technology. The transcription start sites and operon structures were identified throughout the genome. The structure of operons involved in metabolic pathways for acid and solvent production in *C. beijerinckii *8052 were confirmed. Important operons related to chemotaxis/motility, transcriptional regulation, stress response and fatty acids biosynthesis along with others were defined. Twenty previously non-annotated regions were discovered with significant transcriptional activities and 15 genes were identified whose translation start codons were likely mis-annotated. As a consequence, the accuracy of existing genome annotation was significantly enhanced. Moreover, 78 silent genes and 177 putative housekeeping genes were identified based on normalized transcription measurement with the sequence data. More than 30% of the pseudogenes were observed to have significant transcriptional activities during the fermentation process. Strong correlations exist between the expression values derived from RNA-Seq analysis and microarray data or qRT-PCR results. Transcriptome structural profiling in this study provided important supplemental information on the accuracy of annotation of the *C. beijerinckii *genome.

## Methods

### Bacterial culture and fermentation experiment

Laboratory stocks of *C. beijerinckii *8052 spores were stored in sterile H_2_O at 4°C [46]. Spores were heat-shocked at 80°C for 10 min, followed by cooling on ice for 5 min. The heat-shocked spores were inoculated into tryptone-glucose-yeast extract (TGY) medium containing 30 g L^-1 ^tryptone, 20 g L^-1 ^glucose, 10 g L^-1 ^yeast extract and 1 g L^-1 ^L-cysteine at a 1% inoculum level. The TGY culture was incubated at 35 ± 1°C for 12-14 h in an anaerobic chamber under N_2_:CO_2_:H_2 _(volume ratio of 85:10:5) atmosphere. Subsequently, actively growing culture was inoculated into a model solution containing 60 g L^-1 ^glucose, 1 g L^-1 ^yeast extract, and filter-sterilized P2 medium [47,48] in a Sixfors bioreactor system (Infors AG, Bottmingen, Switzerland). Throughout the experiment, oxygen-free nitrogen was flushed through the broth to maintain anaerobiosis. Temperature was controlled at 35 ± 1°C. A stirring at 50 rpm was employed for mixing. During the course of fermentation, samples were collected for cell density and product concentration measurements. For sequencing purpose, RNA samples were taken over the early exponential, late exponential and stationary phases (sample 1-6 at 2, 4.5, 10, 14, 17 and 26.5 h respectively as shown in Figure 1A).

### Culture growth and fermentation products analysis

Culture growth was measured by following optical density (OD) in the fermentation broth at A_600 _using a BioMate 5 UV-Vis Spectrophotometer (Thermo Fisher Scientific Inc., Waltham, MA). ABE, acetic acid, and butyric acid concentrations were quantified using gas chromatography (GC) system as previously described [49].

### RNA isolation, library construction and sequencing

In preparation for RNA isolation, 10 ml cultures were harvested at six time points, and centrifuged at 4,000 × g for 10 min at 4°C. Total RNA was extracted from the cell pellet using Trizol reagent based on manufacture's protocol (Invitrogen, Carlsbad, CA) and further purified using RNeasy minikit (Qiagen, Valencia, CA). DNA was removed using a DNA-*free*™ kit (Ambion Inc., Austin, TX). RNA quality was assessed using a nanochip on a model 2100 bioanalyzer (Agilent Technologies, Santa Clara, CA). RNA concentration was determined with a nanodrop (Biotek Instruments, Winooski, VT). Bacterial 16S and 23S ribosomal RNAs were removed with a MICROBExpress™ kit (Ambion Inc., Austin, TX). The enriched mRNA was converted to a RNA-Seq library using the mRNA-Seq library construction kit (Illumina Inc., San Diego, CA) following manufacturer's protocols. For samples 1 to 6, two samples were pooled and sequenced on one single lane of an eight-lane flow cell with the Genome Analyzer IIx system (Illumina Inc., San Diego, CA). However, sample 6 yielded a poor read quality following the first sequencing. In order to obtain enough sequencing depth, sample 6 was sequenced again using one single lane under otherwise identical conditions. The derived sequence reads were 75 nt long. The overall error rate of the control DNA was < 0.6%. The total number of reads generated from each library is summarized in Table 1.

### Sequence mapping and visualization

The generated 75-nt reads were mapped to the *C. beijerinckii *8052 genome using MAQ, and those that did not align uniquely to the genome were discarded [5,50]. The quality parameter (-q) used in MAQ pileup was set to 30. Each base was assigned a value based on the number of mapped sequence coverage. The coverage plot files were read into Artemis and visualized as sequence coverage profiles over the entire genome [5,9]. Transcription start sites (TSS) were manually identified as described in Passalacqua *et al*. [4]. The region between determined TSS and the annotated translation start site was defined as the 5'-untranslated region (5'-UTR).

### Measurement of gene expression

The quantitative gene expression value, RPKM (reads/Kb/Million), was calculated using custom Perl scripts by normalizing the sequence coverage over the gene length and total unambiguously mapped reads in each library [7,37].

### End-point RT-PCR for operon structure assessment

End-point RT-PCR was performed as described in Passalacqua *et al*. [4]. For each selected co-operonic gene pair, four primers 1F, 1R, 2F and 2R were designed. Primers 1F and 1R amplify a region within gene 1, while 2F and 2R amplify a region within gene 2. When a continuous transcript exists, 1F and 2R amplify across the intergenic region between genes 1 and 2. Therefore, the size of the end-point RT-PCR product will be the same using either cDNA or genomic DNA as templates. The reaction products were visualized on 1.5% agarose gels stained with ethidium bromide.

### Real time qRT-PCR

Quantitative reverse transcription PCR (qRT-PCR) was performed in order to validate the quantification of gene expression level by RNA-Seq. Twenty-five genes were chosen to represent a large range of RPKM values (from ~ 40 to > 18000). Triplicate reactions were performed using Power SYBR green PCR master mix (Applied Biosystems, Carlsbad, CA) on an ABI Prism 7900 HT fast real-time PCR machine (Applied Biosystesms). Detected genes and primer sequences are listed in Additional file 15 Table S11.

### RNA-Seq data accession number

The RNA-Seq sequencing data have been deposited in the NCBI Sequence Read Archive (SRA) under the accession number SRA045799.

## Competing interests

The authors declare that they have no competing interests.

## Authors' contributions

YW, XL, HPB conceived and designed the study. YW and XL performed the experiments. YW, XL and YM analyzed the RNA-Seq data. YW, XL and HPB wrote the manuscript, with input from all authors. All authors discussed the results, read and approved the final manuscript.

## Supplementary Material

Additional file 1 **Circular plots of the reads from all six samples mapping to the *C. beijerinckii *8052 genome**. The outermost and second outermost circles represent CDS on the forward and reverse strands respectively, both of which are colored according to Clusters of Orthologous Groups (COG) functional classification assigned to *C. beijerinckii *8052 annotation. The gold peak and shading area represents greater than the average and lower (in purple). The COG functional classes and corresponding color-coding are as follows (with RGB color model values in the parentheses): Class J, black (0 0 0); Class K, blue (0 0 255); Class L, brown (165 42 42); Class B, dark blue (0 0 139); Class D, chocolate (210 105 30); Class V, cyan (0 255 255); Class T, red (255 0 0); Class M, yellow (255 255 0); Class N, dark green (0 100 0); Class U, grey (128 128 128); Class O, gold (255 215 0); Class C, orange (255 165 0); Class G, light grey (170 170 170); Class E, mid red (255 63 63); Class F, pink (255 192 203); Class H, purple (128 0 128); Class I, violet (238 130 138); Class P, skyblue (135 206 235); Class Q, tan (210 180 140); Class R, darkgrey (100 100 100); Class S, darkred (139 0 0); Not in COGs, green (0 255 0). If one gene belongs to more than one COG classes, the color for that gene was defined by the first class it belongs to as the above order. Click here for file

Additional file 2 **The corresponding Gene ID and ortholog gene in *C. acetobutylicum *ATCC 824 for the genes in *C. beijerinckii *8052 mentioned in the paper (three-letter short names were used in the main text)**. Click here for file

Additional file 3 **TSS and operon structure in *C. beijerinckii *8052 chromosome**. Click here for file

Additional file 4 **Refined genome annotation (in GenBank format) based on the findings from this work and the current *C. beijerinckii *8052 genome annotation in NCBI**. The GenBank file can also be downloaded from https://netfiles.uiuc.edu/blaschek/www/Wang-BMC2011. Click here for file

Additional file 5 **Genes with 5'-UTR length ≥ 100 nt**. Click here for file

Additional file 6 **Putative riboswitches identified using RibEx among the 5'-UTRs over 100 nt**. Click here for file

Additional file 7 **Images of end-point RT-PCR products for 11 selected co-operonic gene pairs on a 1.5% agarose gel**. For each gene pair (1-11, see details in Additional file 8, Table S5) as labeled on the top, the band on the left lane is PCR product using cDNA reverse-transcribed from RNA samples as template, on the right is PCR product using genomic DNA as template. Click here for file

Additional file 8 **Operon structure validation with end-point RT-PCR**. Click here for file

Additional file 9 **Supplemental texts**. Click here for file

Additional file 10 **Potential new genes predicted in non-annotated regions with significant transcriptional activities using GeneMark**. Click here for file

Additional file 11 **Putative silent genes over all the six sampling time points**. Click here for file

Additional file 12 **Putative housekeeping genes (HKGs)**. Click here for file

Additional file 13 **Transcription of the pseudogenes**. Click here for file

Additional file 14 **Summary of the supplementary information provided by RNA-Seq to the current *C. beijerinckii *8052 genome annotation**. Click here for file

Additional file 15 **Genes and primer sequences for qRT-PCR test**. Click here for file
