# Supplementary material for: Single-nucleotide resolution analysis of the transcriptome structure of Clostridium beijerinckii NCIMB 8052 using RNA-Seq
Source: BMC Genomics. 2011 Sep 30;12:479. doi: 10.1186/1471-2164-12-479 (PMC3271303; doi:10.1186/1471-2164-12-479)
Supplement: Additional file 1 — Circular plots of the reads from all six samples mapping to the C. beijerinckii 8052 genome. The outermost and second outermost circles represent CDS on the forward and reverse strands respectively, both of which are colored according to Clusters of Orthologous Groups (COG) functional classification assigned to C. beijerinckii 8052 annotation. The gold peak and shading area represents greater than the average and lower (in purple). The COG functional classes and corresponding color-coding are as follows (with RGB color model values in the parentheses): Class J, black (0 0 0); Class K, blue (0 0 255); Class L, brown (165 42 42); Class B, dark blue (0 0 139); Class D, chocolate (210 105 30); Class V, cyan (0 255 255); Class T, red (255 0 0); Class M, yellow (255 255 0); Class N, dark green (0 100 0); Class U, grey (128 128 128); Class O, gold (255 215 0); Class C, orange (255 165 0); Class G, light grey (170 170 170); Class E, mid red (255 63 63); Class F, pink (255 192 203); Class H, purple (128 0 128); Class I, violet (238 130 138); Class P, skyblue (135 206 235); Class Q, tan (210 180 140); Class R, darkgrey (100 100 100); Class S, darkred (139 0 0); Not in COGs, green (0 255 0). If one gene belongs to more than one COG classes, the color for that gene was defined by the first class it belongs to as the above order. [file 1471-2164-12-479-S1.DOC]

|  |  |
| --- | --- |
| 2 h | 4.5 h |
|  |  |
| 10 h | 14 h |
|  |  |
| 17 h | 26.5 h |

**Figure S1 Circular plots of the reads from all six samples mapping to the *C. beijerinckii* 8052 genome.** The outermost and second outermost circles represent CDS on the forward and reverse strands respectively, both of which are colored according to Clusters of Orthologous Groups (COG) functional classification assigned to *C. beijerinckii* 8052 annotation. The gold peak and shading area represents greater than the average and lower (in purple). The COG functional classes and corresponding color-coding are as follows (with RGB color model values in the parentheses): Class J, black (0 0 0); Class K, blue (0 0 255); Class L, brown (165 42 42); Class B, dark blue (0 0 139); Class D, chocolate (210 105 30); Class V, cyan (0 255 255); Class T, red (255 0 0); Class M, yellow (255 255 0); Class N, dark green (0 100 0); Class U, grey (128 128 128); Class O, gold (255 215 0); Class C, orange (255 165 0); Class G, light grey (170 170 170); Class E, mid red (255 63 63); Class F, pink (255 192 203); Class H, purple (128 0 128); Class I, violet (238 130 138); Class P, skyblue (135 206 235); Class Q, tan (210 180 140); Class R, darkgrey (100 100 100); Class S, darkred (139 0 0); Not in COGs, green (0 255 0). If one gene belongs to more than one COG classes, the color for that gene was defined by the first class it belongs to as the above order.
