# Supplementary figures and images for: Single-nucleotide resolution analysis of the transcriptome structure of Clostridium beijerinckii NCIMB 8052 using RNA-Seq
Source: BMC Genomics. 2011 Sep 30;12:479. doi: 10.1186/1471-2164-12-479 (PMC3271303; doi:10.1186/1471-2164-12-479)

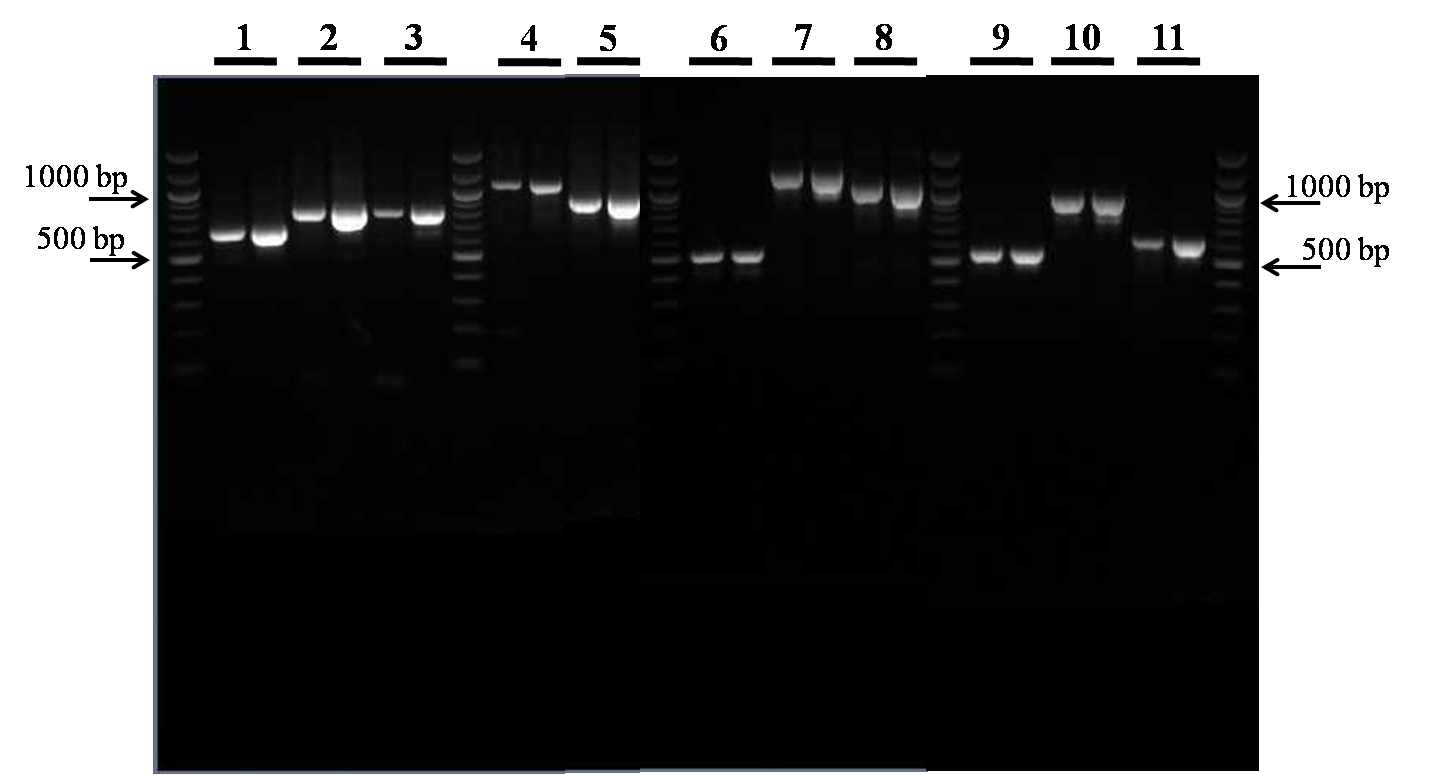

Supplement: Additional file 4 — Refined genome annotation (in GenBank format) based on the findings from this work and the current C. beijerinckii 8052 genome annotation in NCBI. The GenBank file can also be downloaded from https://netfiles.uiuc.edu/blaschek/www/Wang-BMC2011. [file 1471-2164-12-479-S4.TIFF]
