# Supplementary material for: Single-nucleotide resolution analysis of the transcriptome structure of Clostridium beijerinckii NCIMB 8052 using RNA-Seq
Source: BMC Genomics. 2011 Sep 30;12:479. doi: 10.1186/1471-2164-12-479 (PMC3271303; doi:10.1186/1471-2164-12-479)
Supplement: Additional file 8 — Operon structure validation with end-point RT-PCR. [file 1471-2164-12-479-S8.DOC]

**Table S5 Operon structure validation with end-point RT-PCR.**

| Sample | Gene pair^*^ | Strand | Intergenic distance (bp) | Expected RT-PCR product length (bp) |
| --- | --- | --- | --- | --- |
| 1 | 0203-0204 | + | 144 | 659 |
| 2 | 0341-0342^**^ | + | 7 | 811 |
| 3 | 0596-0597 | + | 71 | 811 |
| 4 | 0598-0599 | + | 432 | 1082 |
| 5 | 0674-0675 | - | 97 | 910 |
| 6 | 0837-0838 | + | 142 | 525 |
| 7 | 1136-1137 | + | 440 | 1235 |
| 8 | 2599-2600 | - | 394 | 1057 |
| 9 | 3256-3257 | - | 123 | 557 |
| 10 | 3832-3833 | + | 91 | 976 |
| 11 | 4318-4319 | - | 150 | 634 |

^*^Numbers are ‘Cbei’ numbers from *C. beijerinckii* NCIMB 8052 genome annotation.

^**^Highly likely operon gene pair used as a positive control.
