# Supplementary material for: Single-nucleotide resolution analysis of the transcriptome structure of Clostridium beijerinckii NCIMB 8052 using RNA-Seq
Source: BMC Genomics. 2011 Sep 30;12:479. doi: 10.1186/1471-2164-12-479 (PMC3271303; doi:10.1186/1471-2164-12-479)
Supplement: Additional file 9 — Supplemental texts. [file 1471-2164-12-479-S9.DOC]

**Supplemental texts**

**For testing and enhancing the current *C. beijerinckii* 8052 genome annotation:**

**Non-annotated regions with significant transcriptional activity:** Cbei_0576-0577, Cbei_1425-1426, Cbei_1460-1461, Cbei_1511-1512, Cbei_1572-1573, Cbei_1607-1608, Cbei_1748-1749, Cbei_2042-2043, Cbei_2151-2152, Cbei_2344-2345, Cbei_2395-2396, Cbei_2480-2481, Cbei_3091-3092, Cbei_3110-3111, Cbei_3202-3203, Cbei_3285-3286, Cbei_3362-3363, Cbei_3591-3592, Cbei_4206-4207 and Cbei_4988-4989.

These regions were tested for potential new genes with GeneMark (<http://exon.gatech.edu/genemark/>). See Additional file 10, Table S6 for details.

**Genes with potential incorrect annotated translation start sites (potential mis-annotations):** Cbei_0427, Cbei_0469, Cbei_0574, Cbei_0889, Cbei_0933, Cbei_1291, Cbei_1377, Cbei_1404, Cbei_1407, Cbei_1716, Cbei_1787, Cbei_3528, Cbei_3767, Cbei_4000 and Cbei_5042.
