# Supplementary material for: Single-nucleotide resolution analysis of the transcriptome structure of Clostridium beijerinckii NCIMB 8052 using RNA-Seq
Source: BMC Genomics. 2011 Sep 30;12:479. doi: 10.1186/1471-2164-12-479 (PMC3271303; doi:10.1186/1471-2164-12-479)
Supplement: Additional file 15 — Genes and primer sequences for qRT-PCR test. [file 1471-2164-12-479-S15.DOC]

**Table S11 Genes and primer sequences for qRT-PCR test.**

| No. | Gene ID | Strand | Forward primer (5'-3') | Reverse primer (5'-3') | Size of amplicon (bp) | Gene product description |
| --- | --- | --- | --- | --- | --- | --- |
| 1 | Cbei_0204 | + | A T A T G G C A A G G C T G T A G G A A A A | A A G G T C T G G A A C A A G T G T T G G T | 235 | butyrate kinase |
| 2 | Cbei_0325 | + | T C C A A T G G G A C C T T T A G C T T T A | T C C T T T T C C T G A T T T T C T T C C A | 163 | 3-hydroxybutyryl-CoA dehydrogenase |
| 3 | Cbei_0411 | + | T G G A C C A T T C C A T G C A A C T A | T G C T A A G C T T T G A G C A G C A A | 106 | acetyl-CoA acetyltransferase |
| 4 | Cbei_0599 | + | T T A T C G C A G G A A A C T G G A A A A T | C T T C T C C T G T G A A A G C T C C A C T | 216 | triosephosphate isomerase |
| 5 | Cbei_0602 | + | A G G T A T T G G C G A T G A A G G T G | A A G C T T C C A A C C T T C C C A A T | 295 | phosphopyruvate hydratase |
| 6 | Cbei_0837 | + | C C T G A A G C T C C A A C T C A T T T C T | C T G G T G G C C A C T G T A A A C T T C | 244 | histidine triad (HIT) protein |
| 7 | Cbei_0838 | + | T T G G A G A A A A C G A A A C A C T T G A | T T C T T A A C G C T T G G C T T T T C A | 118 | 30S ribosomal protein S21 |
| 8 | Cbei_1055 | + | G C A A C A G C A T T C A A T G G A G A | T C T T A G C C A T G C T T C C T G C T | 217 | phosphoribosylaminoimidazole-succinocarboxamide synthase |
| 9 | Cbei_1105 | + | A A T A T T G A A C A T A C A A T G C A A T T T G A | T G C T C C G T T G T A A T T A G T G A T G T A A | 174 | hypothetical protein |
| 10 | Cbei_1480 | - | A T A T G T C C G A A G C C T T C C A G T | A C C A T C T C C A T A G C T G G G T T T | 122 | LytTR family two component transcriptional regulator |
| 11 | Cbei_1712 | + | T G A A G T T G G A G T A C C A G C T C A T | T G A C C T C T T C C C C A T G C T A C | 198 | response regulator receiver protein |
| 12 | Cbei_1797 | - | A G C T G G C G G A G A T G C T A G T | G C A C C T C A G G G T C C A T T C T | 101 | aspartate-semialdehyde dehydrogenase |
| 13 | Cbei_1960 | + | T G A T T C A G A T G A A A T T G G T G T T G | C T C C A T C A C C T T C A A T T T T T G A G | 224 | 2-dehydropantoate 2-reductase |
| 14 | Cbei_2428 | + | A C A G A T G G A A C A T C A T T G C T T G | A T C A A A T A A A T G C G C T C C A A G T | 159 | peptidase T |
| 15 | Cbei_2583 | + | C A A G A T G C A G C T G A T T T G G A | G G G T C T T C C T C T G G T T C C T C | 224 | hypothetical protein |
| 16 | Cbei_2584 | + | T G C A G T T G C A T T C A C A G G A | A C A G C A G A A A T C G C C A C A G | 163 | hypothetical protein |
| 17 | Cbei_2599 | - | G C T A C G T C A T T T C C A A A A C C A | C T T C T G T T G C A T T G C T T G T G A | 202 | cysteine desulfurase family protein |
| 18 | Cbei_2600 | - | C T C A C C A C G T C C T G G T A T T G T | C G C T T C T T A A T T C G C A A T C T G | 164 | hypothetical protein |
| 19 | Cbei_3257 | - | C G C G C C T T A A C A G A A T C A A | T T C G C C A C T C T T C A A C C T G | 130 | rubrerythrin |
| 20 | Cbei_3650 | - | T C A C T T T G C A T G T A A G G A T G A G A T | T T T A T C A A T C G C T G A C T G T T G A T T | 112 | TetR family transcriptional regulator |
| 21 | Cbei_3833 | + | T C C C C A C A A G G A A C A C T G A | T T T G C A G C C A T T G C C A T A | 257 | 3-oxoacid CoA-transferase, A subunit |
| 22 | Cbei_3834 | + | T G T T G C T G T T C T T G G T G C T C | T T G C G C C T A T T G C T A A A T C C | 116 | 3-oxoacid CoA-transferase, B subunit |
| 23 | Cbei_3835 | + | C T G C A A C A A T G G G A T A T A A G C A | A G T C C A A G C A C C G T G A A T A G T T | 170 | acetoacetate decarboxylase |
| 24 | Cbei_4319 | - | A G G A G C G G A A G T A A G T G T G A A G | T T T A G T G G C A T C T A C T G C T G G A | 103 | flavodoxin |
| 25 | Cbei_5079 | - | A G G A G T T T G C C T C A A A G C A T A G | A T A G G T T A C C C C T C C A G C T C T C | 243 | replicative DNA helicase |
